# A. V. M. A.

**Published:** 1900-11

**Authors:** 


### A. V. M. A.

*Executive Committee*, 1900–1901.

Dr. R. R. Bell, Chairman, Brooklyn, N. Y.; Dr. D. E. Salmon, Washington, D. C.; Dr. M. H. Reynolds, St. Anthony's Park, Minn.; Dr. C. A. Cary, Auburn, Ala.; Dr. W. L. Williams, Ithaca, N. Y.; Dr. G. A. Johnson, Sioux City, Iowa; Dr. W. Horace Hoskins, Philadelphia, Pa.

*Ex-officio.*

Dr. Tait Butler, President, Indianapolis, Ind.; Dr. S. Brenton, Vice-President, Detroit, Mich.; Dr. W. H. Dalrymple, Vice-President, Baton Rouge, La.; Dr. J. F. Winchester, Vice-President, Lawrence, Mass.; Dr. F. Torrance, Vice-President, Winnipeg, Manitoba; Dr. E. M. Knowles, Vice-President, Helena, Montana; Dr. S. Stewart, Secretary, Kansas City, Kan.; Dr. W. Herbert Lowe, Treasurer, Paterson, N. J.

